# Genetic and phenotypic characterization of the native rabbits in Middle Egypt

**DOI:** 10.14202/vetworld.2018.1120-1126

**Published:** 2018-08-14

**Authors:** El-Sayed Mahfouz Abdel-Kafy, Sahar Saad El-Din Ahmed, Amira El-keredy, Neama Ibrahim Ali, Sherif Ramadan, Ahmed Farid

**Affiliations:** 1 Department of Rabbit Breeding Research, Animal Production Research Institute, Agricultural Research Centre, Dokki, Giza, Egypt; 2 Department of Cell Biology, Division research of Genetic Engineering and Biotechnology, National Research Centre, Giza, Egypt; 3 Department of Genetics, Faculty of Agriculture, Tanta University, Tanta, Egypt; 4 Department of Animal Wealth Development, Faculty of Veterinary Medicine, Benha University, Egypt

**Keywords:** microsatellite markers, middle of Egypt, mitochondrial DNA, native breed, phenotypic, rabbit

## Abstract

**Aim:**

Native rabbits in smallholder system are considered as important genetic resources, and the present study was aimed to study the genetic and phenotypic characterization and detection of the maternal origin of the native rabbit populations located at the Middle of Egypt.

**Materials and Methods:**

A survey of native rabbit populations was conducted in three governorates (Fayum [FY], Beni Suef [BN], and El Menia [MN]). The phenotypic characterization of rabbits included the profile body of the head, ears, eyes, neck, and legs and the coat colors. The blood samples were collected for genetic characterization based on mitochondrial (cytochrome *b*) and the microsatellite markers.

**Results:**

The phenotypic characterization of the body parts in the three populations was almost similar. The body weight of the mature rabbits in MN Government was significantly heaviest, and the measurements for the main body parts (body length, chest circumference, and abdominal girth) were the highest compared to the two populations. The results of mitochondrial (cytochrome *b*) analysis revealed that the rabbits from the three governments belonged to lineage A except one animal was recorded as lineage G from MN’s rabbit population. The results of the microsatellite markers revealed that the genetic diversity between the three populations showed genetic interferences; however, a closer genetic relationship was observed between BN and MN than FY. The majority of the genetic diversity was the individual variability.

**Conclusion:**

The mitochondrial lineage A is the major lineage in rabbit populations in the area of the Middle Egypt understudy. The genetic populations’ structure is the interferences among the three populations. A large-scale survey should be done on native rabbit populations for the sustainable management and conservation of the local breeds’ genetic resources.

## Introduction

Native rabbit breeds by smallholder under low-input systems consider as important genetic resources because of their adaptation to harsh environmental conditions and their tolerance to a wide range of diseases. Accordingly, local breeds should be protected against any threat factors as a priority of sustainable management [1].

In Egypt, native rabbits were used early to develop many breeds such as Giza White, Baladi Red, Baladi Black, and Baladi White by selection and/or different crosses between native and exotic rabbit breeds, although the importance of the native breeds, few studies have been done concerning these rabbits [2].

Phenotypic and genetic characterizations of the native rabbits are essential studies for the identification and improvement of the breeding programs and to help for their conservation [3]. The population genetic structure is the result of many factors such as geographical and ecological factors which may cause variation and division of the populations. Moreover, the genetic drift, gene flow, and the balance between them can also result in population genetic structure [4,5]. Genetic studies using mitochondrial DNA and microsatellite markers reported as essential genetics tools to explain the population structure at different temporal and spatial scales and the genetic flow among individuals within and between populations [6-9].

The purposes of this study were (1) to measure the phenotypic characterization of native rabbit populations in three governorates (Fayum [FY], Beni Suef [BN], and El Menia [MN]) at the Middle Egypt region through evaluation of external body measurements, (2) to identify the maternal origin using the mitochondrial DNA (cytochrome *b*), and (3) to evaluate the genetic diversity between the populations using microsatellite markers.

## Materials and Methods

### Ethical approval

Experiments were carried out in accordance with the guidelines laid down by the Institutional Animal Ethics Committee and in accordance with the local laws and regulations.

### Animals

**Figure-1:**
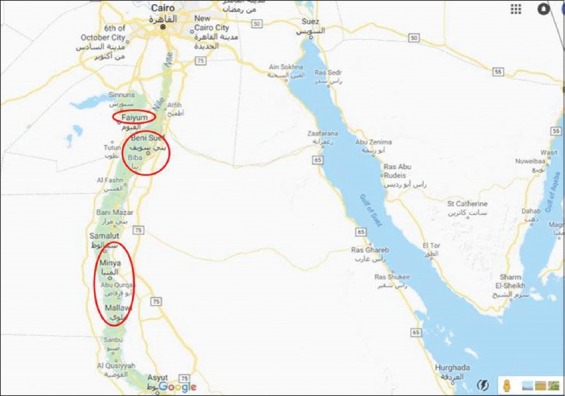
The geographical locations of Fayum, Beni Suef, and El Menia Governorates in Egypt.

A survey of the native rabbit populations was conducted in three governorates, FY, BN, and MN in a period from December 2017 to February 2018 (Figure-1). The phenotype data and blood samples of Native Middle Egypt rabbits (NMERs) were collected randomly from six localities of the three governorates. The phenotypic characterization of NMER was made according to Khalil [10] and included the profile body of the head, ears, eyes, neck, and legs and the coat colors. The morphological characteristics of NMER populations were taken by body weight and body dimension measurements on 83 mature rabbits. The body length was measured from atlas to the first coccygeal vertebrae, while the chest circumference was measured behind the shoulder blades and the abdominal circumference at the level of the seventh lumbar vertebrae. The ear width was from the outside to another side of the ear. Ear length was taken from the bottom to the top of the ear, and foot length was from behind the foot to start the fingers.

### Sample collection and DNA extraction

A total of 83 blood samples from three rabbits populations: FY (n=31), BN (n=31), and MN (n=21) were used for genetic characterization. Blood samples were collected into 5 mL Vacutainer tubes containing EDTA as an anticoagulant and stored at 4°C until molecular analyses were performed. DNA was extracted from rabbit blood samples using Chelex 100^®^ according to a slight modification of a protocol of Welsh and McClelland [11].

### Mitochondrial genotyping

Two primers of the cytochrome *b* gene were used according to Branco *et al*. [12] (CBF: 5’-ATGACCAACATTCGCAAAACC-3’ and CBR: 5’-TGTCTCAGGGAGAACTATCTCC-3’). The polymerase chain reaction (PCR) was performed in a final volume of 25 μL containing: 200 μM of dNTPs, 0.2 μM of each primer, 1 U *Taq* polymerase (H. T. Biotechnology), and 1 μL of DNA extract. The PCR program consisted of 4 min of denaturation at 94°C, followed by 40 cycles of 1 min at 94°C, 1 min at 55°C, and 1 min 30 s at 72°C, plus a final extension of 10 min at 72°C. The PCR products were digested with *AluI* restriction enzymes (Promega, France), following the manufacture instruction. The PCR products and 100-bp DNA ladder (Promega Corporation, France) were electrophoresed on 2% agarose gel (Invitrogen Ultrapure™ Agarose^®^, Carlsbad, USA). The amplified products were visualized in the gel documentation apparatus, photographed and analyzed according to the number and the size of fragments resulted from restriction enzyme digestion.

### Microsatellite genotyping

Eight microsatellite markers were used for genotyping analysis (*Sat2, Sat3, Sat5, Sat7, Sat8, Sat12, Sat13, and Sat16*) [13-15]. PCR reactions were performed in a final volume of 15 μL that contained 200 μM dNTPs, 0.2-0.4 μM of each primer, 0.325 U *Taq* polymerase (H. T. Biotechnology), and 0.5 μL of DNA extract. PCR programs involved 5 min of initial denaturation at 95°C, followed by 30 cycles of 30 s at 95°C, 30 s between 55°C and 60°C, and 30 s at 72°C, followed by a final extension step of 10 min at 72°C. The PCR products were separated on denaturing electrophoresis in 3% polyacrylamide gels containing urea, and bands were visualized by rapid silver staining [16].

### Statistical analysis

Least square means for body weight and body dimension measurements were estimated using the GLM procedure of SAS version 9.1.3 (SAS Institute Inc., Cary, NC, USA). The model used was Yij=µ+Pi+eij, where Yij=any observation of rabbit within i^th^ populations (P), µ=overall mean, Pi=the effect of the populations, i=1, 2, and 3, and eij=the random error. Significant differences between the populations were defined by Duncan test.

Genetic diversity was evaluated by calculating the observed and effective number of alleles (*N_o_* and *N_e_*), heterozygosity observed and expected (*H_o_* and *H_e_*) using GENALEX software version 6.0 [17]. Polymorphic information content (*PIC*) was assessed using CERVUS software version 3 [18]. Pairwise *F_ST_* [19] in addition to F-statistics (*F_IS_*, *F_ST_*, and *F_IT_*) across the three studied populations were calculated using the GENEPOP software version 3.4 [20]. Genetic distances among the three studied populations were evaluated by Reynolds genetic distance [21]. A neighbor-joining (NJ) phylogenetic tree was constructed based on the Reynolds genetic distance [22]. These processes were conducted using POPULATIONS version 1.2.30 software; pairwise comparisons of the 20 runs of each K value were done with 20 permutations using CLUMPP software [23].

The STRUCTURE software was investigated for the analysis of the genetic structure and clustering the three studied rabbit populations, 20 runs for each different value of *K* with 50,000 iterations following a burn-in period of 50,000. The clustering pattern with the highest *H* value was graphically displayed for the selected *K* value using DISTRUCT software [24].

## Results and Discussion

### Phenotypic characterization

**Figure-2:**
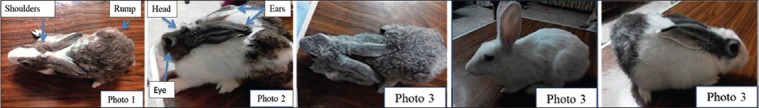
The native Middle Egypt rabbit phenotypes.

The body profile showed that the base in the rump was wide, round, and full appearance while at shoulders apart are low and narrow, but they are sturdy (Figure-2: Photo-1). The head was between medium and small in size, but it was long and narrow in proportion. The profile of the head was sloped from the base of the ears down to the nose in a line which was almost straight. The ears were relatively long. The eyes were large and protruding (Figure-2: Photo-2). The neck was short but visible. Legs were strong, straight with a medium length. The coat colors included different models: Grayish, brown, and white (Figure-2: Photo-3a and b) and sometimes gray or red sprinkled throughout the coat. Another model, the underside of the body, was pale-gray or black or brown, while the upper side of the body was white (Figure-2: Photo-3c). The phenotypic results revealed that the NMER in the three populations was almost similar and very close to the rabbit in Malta [25].

### Body weight and body dimensions

The size of the NMER in the three populations was small to medium. The mean of the body weights, body length, chest circumference, and abdominal girth of NMER is shown in Table-1. The body weights of FY and BN governments were closer to the Dwarf rabbit in Italy which ranged from 1953 to 1850 g [26], while the body length was medium and the chest circumference was similar to that of the local rabbit breeds in Lebanon [27]. The mean of the body weights of the mature rabbits in MN government was significantly heaviest, and the measurements for the main body parts (body length, chest circumference, and abdominal girth) were highest among the three studied populations. The phenotypic characterization of the BN government was significantly measured less than other populations.

**Table-1:** Measurements of body weights and body dimensions of NMER rabbit populations.

Geographical location	Weight (g)	Body length (cm)	Chest circumference (cm)	Abdominal girth (cm)	Ear width (cm)	Ear length (cm)	Foot length (cm)
FY	1691.9±55.8^b^	32.8±0.35^ab^	24.2±0.30^b^	24.8±0.48^b^	5.72±0.04	9.80±0.08	6.62±0.08
BN	1538.6±55.8^b^	32.5±0.35^b^	23.0±0.30^b^	22.8±0.48^b^	5.80±0.04	10.17±0.08	6.51±0.08
MN	2062.9±68.2^a^	35.2±0.41^a^	27.4±0.36^a^	29.9±0.55^a^	6.14±0.06	9.91±0.12	6.56±0.13
Mean±SE	1662.9±62.8	32.9±0.38	24.0±0.33	24.5±0.52	5.7±0.05	9.9±0.09	6.5±0.09

Means with different superscript letters are significantly different. NMER=Native Middle Egypt rabbit, BN=BeniSuef, MN=El Menia, FY=Fayum, SE=Standard error

### Genetic characterizations

#### Mitochondrial analysis

**Figure-3:**
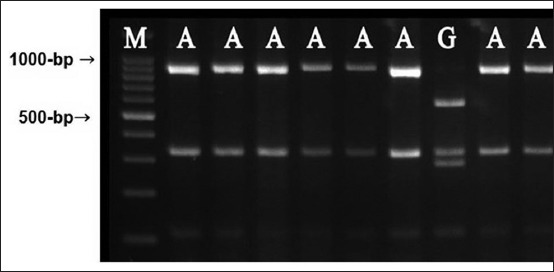
Genotypic patterns of cytochrome *b* in the native Middle Egypt rabbits, M is a 100-bp DNA ladder.

The results of the mitochondrial cytochrome *b* revealed that the overall NMERs carried lineages A, while lineage G was found as single case from MN (Figure-3). The current study considered as the first detection of the maternal origin in the native Egyptian rabbit populations. The data generated here provide valuable information related to the common ancestor of the rabbits between many places in Middle Egypt according to cytochrome *b* analysis. Existence of mitochondrial lineage A as the major lineage in Egyptian rabbit populations may support the theory that rabbits were transferred from southwest of the Iberian Peninsula to Egypt and North of Africa zone, where lineage A is predominant in the subspecies *Oryctolagus cuniculus algirus* inhabiting the Southwest of the Iberian Peninsula and lineage B is predominant in *O. cuniculus* inhabiting the northeast of the Peninsula [28].

#### Microsatellites analysis

The genetic diversity of the NMER populations shows that the alleles detected across eight microsatellite loci in the three studied populations were 46 (Table-2). The number of observed alleles (*N_o_*) ranged between 3.64 and 10 with an average 6.125. The majority of the markers were characterized by high allelic values per locus except for *Sat2* and *Sat7*. The polymorphism information content per locus *(PIC)* index which is revealed the degree of microsatellite loci polymorphism recorded higher values exceeding 0.5 in all markers except for *Sat2* (0.326) and *Sat7* (0.474). The total population heterozygosity (*H_e_*) of microsatellite loci ranged between 0.340 and 0.878 with an average 0.705. These results are relatively higher than the study of Xin-Sheng *et al*. [29] in Wan line Angora rabbit which was 4.5 for *N_o_*, 0.680 for *H_e_*, and 0.642 for *PIC*. However, our results were lower than reported by El-Aksher *et al*. [30] whose recorded average values for *H_o_* (0.650), *H_e_* (0.800), and *PIC* (0.760) across four Egyptian rabbit populations different than the rabbit populations understudy. The studied microsatellite markers deviated significantly from Hardy–Weinberg equilibrium (p<0.001) except *Sat2* and *Sat7*. The results suggested that the rabbits under study might be a non-random mating or undergo certain kind of selection for some favorable traits.

**Table-2:** Observed (*N_o_*) and effective (*N_e_*) numbers of alleles per microsatellite marker and observed (*H_o_*) and expected (*H_e_*) heterozygosity and PIC for each locus across all three studied rabbit populations.

Marker	Allele range (bp)	*N_o_*	*N_e_*	*H_o_*	*H_e_*	*PIC*	*F_IS_*	*F_IS_*	*F_IS_*	HWE
Sat2	233-251	3.667	1.510	0.374	0.340	0.326	−0.121	−0.093	0.025	NS
Sat3	146-166	7.333	5.396	0.582	0.830	0.807	0.285	0.301	0.022	***
Sat5	199-263	5.667	4.626	0.620	0.792	0.766	0.202	0.216	0.017	***
Sat7	182-199	4.000	2.112	0.648	0.533	0.474	−0.238	−0.221	0.014	NS
Sat8	134-158	5.667	3.533	0.695	0.726	0.683	0.024	0.038	0.014	***
Sat12	130-146	5.000	3.515	0.587	0.724	0.726	0.173	0.241	0.082	***
Sat13	96-158	7.000	4.979	0.685	0.813	0.833	0.142	0.193	0.059	***
Sat16	96-158	10.667	7.580	0.759	0.878	0.899	0.119	0.158	0.044	***
Mean±SE	6.125±0.333	4.156±0.566	0.619±0.062	0.705±0.019	0.689±0.026	0.073±0.062	0.104±0.064	0.034±0.009

*N_o_*=Number of observed alleles, *H_o_* and *H_e_*=Mean observed and expected heterozygosity. *PIC*=Mean polymorphism information content per locus, HWE=Hardy–Weinberg equilibrium, SE=Standard error. *p<0.05, **p<0.01, ***p<0.001, NS=Non-significant

### Genetic diversity within population

The mean value of *N_o_* and effective *N_e_* numbers of alleles, observed (*H_o_*) and expected (*H_e_*) heterozygosities, and the fixation coefficient of an individual within a subpopulation (*F_IS_*) are shown in Table-3. The highest value of *N_o_* (6.625) and *N_e_* (4.327) was recorded for FY population, while the lowest values (*N_o_* = 5.625 and *N_e_* = 4.008) were recorded for MN rabbit population. BN and MN populations showed higher expected heterozygosity (*H_e_* = 0.715 and 0.705, respectively) than FY population (*H_e_* = 0.693). The higher observed heterozygosity in the two populations could be attributed to several crosses due to human activities such as exchanging and marketing between different villages. The result could be supported by the higher performance traits of the body weight and dimensions observed in MN population (Table-1) which might be a result of hybrid vigor due to several mating and crosses.

**Table-3:** Observed (*N_o_*) and effective (*N_e_*) numbers of alleles and mean observed (*H_o_*) and expected (*H_e_*) heterozygosities and fixation coefficient of an individual within a subpopulation (*F_IS_*) per population rabbits.

Geographical location	N	*N_o_*±SE	*N_e_*±SE	*H_o_*±SE	*H_e_*±SE	F_IS_±SE
FY	31	6.625±1.322	4.327±0.930	0.625±0.058	0.693±0.072	0.028±0.101
BN	31	6.125±0.515	4.134±0.644	0.577±0.041	0.715±0.055	0.161±0.066
MN	21	5.625±0.653	4.008±0.576	0.655±0.048	0.705±0.067	0.024±0.041
Total		6.125±0.505	4.156±0.405	0.619±0.028	0.705±0.036	0.071±0.043

Number of individuals (N), number of allele (*N_o_*), of effective number of allele (*N_e_*), observed heterozygosity (*H_o_*), and expected heterozygosity (*H_e_*) of local Middle Egypt rabbit populations. BN=BeniSuef, MN=El Menia, FY=Fayum

The results of the mean number of the observed alleles were *N_o_* = 6.125 in NMER which is lower than that observed in another Egyptian rabbit populations (*N_o_* = 6.75) [30]. The value of *F_IS_* among the three rabbit populations was 0.071 which is relatively lower (0.172) than that reported in the Spanish rabbit populations by Grimal *et al*. [31]. On the other hand, the observed heterozygosity of the NME populations recorded higher values compared to what have been reported in Middle Egyptian native rabbit breeds by Abdel-Kafy *et al*. [4], while it was closer to the values of Tunisian’s rabbits (*H_o_* ranging from 0. 365 to 0.482) [32] and Spanish breeds (*H_o_* ranging from 0.36 to 0.48) [31]. The variation between the results of this study and Abdel-Kafy *et al*. [4] is probably due to the sampling process, where they collected from eight geographical locations very near to each other, and each location was considered as a population, while in this study, the samples of each governorate were considered as a one population.

### Genetic diversity between populations

**Figure-4:**
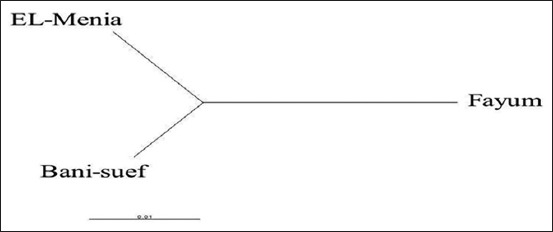
Neighbor-joining population phylogenetic tree for the three studied rabbit populations.

The NJ phylogenetic tree of the investigated rabbit populations showed the close relationship between MN and BN than FY (Figure-4). This could be attributed to the high connection between MN and BN governorates because of the short geographical distance and no natural barriers between them (Figure-1).

The pairwise *F_ST_* values among the three populations were ranged from 0.024 to 0.028. The *F_ST_* values between populations indicated a generally low level of genetic differentiation. The highest *F_ST_* value (0.028) was recorded between MN and FY, while the lowest value (0.024) was between BN and MN government (Table-4). Overall, the genetic distance between the three populations was small which suggested that the three populations were closely related and had a common ancestor. Comparing with the previous studies, the genetic distance between the three populations was lower than that reported for Tunisian rabbit populations [32].

**Table-4:** Reynolds genetic distances (above diagonal) and pairwise *F_ST_* (below diagonal) among the three studied rabbit populations.

Population	FY	BN	MN
FY		0.095	0.09
BN	0.026		0.083
MN	0.028	0.024	

BN=BeniSuef, MN=El Menia, FY=Fayum

**Figure-5:**
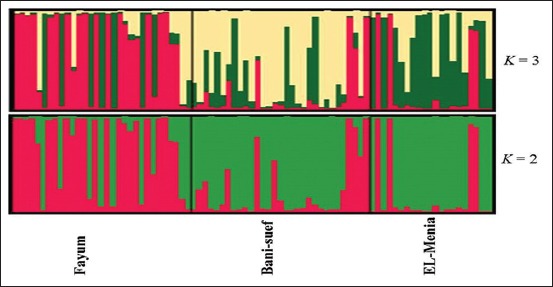
Structure clustering of the three native Middle Egypt rabbit populations obtained for *K*=2 and 3. The *K* is the cluster number.

The investigated population structure using the Bayesian approach revealed that the most structural clustering pattern was shown at the number of clusters (*K*) = 2 where the FY population incompletely separated in the first cluster, while BN and MN clustered together showing admixed mosaic cluster with high interferences between them (Figure-5). In the case of *K*=3, all populations were the interferences and not detected in separate clusters. This result was confirmed by individual phylogenetic tree of the investigated 83 rabbits (Figure-4). This finding could be related to the geographical location of FY Governorate away in the Northeast of the Nile River. Moreover, the BN and MN governorates are located on the river Nile where the human activities like marketing can also shape the rabbit population structure as the rabbit genetic diversity in its native distribution is the result of an ensemble of factors, from geographical and ecological, to behavioral and molecular, that hierarchically interact through time and space [32].

## Conclusion

The phenotypic characterization of the NMER populations showed that the body weights, body length, chest circumference, and abdominal girth of MN rabbit population recorded the highest values among the three populations under the study. The major lineage in the three populations was lineage A. The genetic diversity between the three populations showed a closer genetic relationship between BN and MN rabbit populations than FY, while the result of the genetic distance between the three populations was small which suggested that the three populations were closely related and had a common ancestor. The majority of the genetic diversity was explained by individual variability. A large-scale survey should be done on native rabbit populations for developing sustainable genetic improvement and conservation programs of the local breeds’ genetic resources.

## Authors’ Contributions

EMA and SSA planned and designed the study. AE, NIA, SR, and AF collected the blood samples and provided the help for statistical analysis. NIA, AE, and SSEA carried out the laboratory work. SR and SS provided the help for the data analysis and interpretation of the laboratory results. EMA drafted the manuscript. All authors participated in the revision of the manuscript. All authors read and approved the final manuscript.
